# Immunogenicity and safety of the BBIBP‐CorV vaccine in patients with autoimmune inflammatory rheumatic diseases undergoing immunosuppressive therapy in a monocentric cohort

**DOI:** 10.1002/iid3.858

**Published:** 2023-05-16

**Authors:** Batool Zamani, Amin Moradi Hasan‐Abad, Ahmad Piroozmand, Mahsa Dehghani, Maryam Arfaatabar, Hossein Motedayyen

**Affiliations:** ^1^ Autoimmune Diseases Research Center Kashan University of Medical Sciences Kashan Iran; ^2^ Department of Medical Laboratory Sciences, Kashan Branch Islamic Azad University Kashan Iran

**Keywords:** adverse events, BBIBP‐CorV vaccine, disease activity, immunogenicity, patients with AIIRD

## Abstract

**Introduction:**

Vaccination plays a fundamental role in mastering the COVID‐19 pandemic and protecting vulnerable groups. Persons with autoimmune inflammatory rheumatic diseases (AIIRD) requiring immunosuppressive therapies are prioritized for vaccination. However, data concerning immunogenicity and safety of the BBIBP‐CorV vaccine in immunosuppressed patients are not found. This study presents data on the efficacy and safety of the BBIBP‐CorV vaccine in immunosuppressed patients compared to healthy controls.

**Methods:**

Study population consisted of 100 healthy controls and 100 patients with AIIRD. Vaccination was performed according to national guidelines with the BBIBP‐CorV vaccine. SARS‐CoV‐2 neutralizing antibody titers were quantified by enzyme‐linked immunosorbent assay before initial vaccination and 1–3 months after secondary vaccination. Adverse events were assessed before study initiation and 7 days after the second dose. Disease activity was studied before entering the study and 3–8 weeks after the second dose.

**Results:**

Vaccination‐induced positive immunogenic response rates and SARS‐CoV‐2 neutralizing antibody titers were significantly lower in the AIIRD patients than healthy subjects (*p* < .05). There are significant differences in neutralizing antibody titers among patients suffering from rheumatoid arthritis (RA), systemic lupus erythematosus (SLE), systemic sclerosis, and ankylosing spondylitis (*p* < .01–.05). The rates of seropositive vaccine responses were similarly distributed across all diseases. Healthy and AIIRD individuals had a similar profile in adverse events. No significant difference was observed in SARS‐CoV‐2 antibody titers between subjects suffering from side effects and those who did not have. SARS‐CoV‐2 neutralizing antibody levels were significantly higher in subjects with a history of COVID‐19 infection than seronegative individuals (*p* < .01–0.05). Seropositive subjects had a significant increase in the percentage of vaccine‐related adverse events compared to seronegative persons (*p* < .05). Despite a minor change in the disease activity of patients with RA and SLE, disease activity indices were overall stable in the AIIRD patients.

**Conclusion:**

These findings revealed that the BBIBP‐CorV vaccine is effective in the development of neutralizing antibodies in immunosuppressed patients without considerable reactogenicity or induction of disease flares.

## Graphical abstract

Vaccination plays a fundamental role in mastering the COVID‐19 pandemic and protecting vulnerable groups. Persons with autoimmune inflammatory rheumatic diseases (AIIRD), on immunosuppressive therapies are prioritized for vaccination. However, data concerning immunogenicity and safety of the BBIBP‐CorV vaccine in immunosuppressed patients are not found. This study presents data on the efficacy and safety of the BBIBP‐CorV vaccine in immunosuppressed patients compared to healthy controls. Our findings revealed that the BBIBP‐CorV vaccine is effective in the development of neutralizing antibody in immunosuppressed patients without considerable reactogenicity or induction of disease flares.

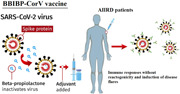

## 1 INTRODUCTION

The SARS‐CoV‐2 provoked COVID‐19 pandemic triggered intense global R&D activity to develop an immunogenic and effective vaccine against the disease (7). On January 11, 2020, the determination of the genetic sequence of SARS‐CoV‐2 has urged the development and authorization of COVID‐19 vaccines. The first‐in‐human clinical trial of COVID‐19 vaccine candidate was performed on March 16, 2020.^1^

Patients with autoimmune inflammatory rheumatic diseases (AIIRD) are generally susceptible to infections and experience worse outcomes and severe course of COVID‐19 disease, due perhaps to extensive treatment with immunosuppressive drugs.^2^ These findings emphasize the importance of SARS‐CoV‐2 vaccination in patients with AIIRD.

Until now, numerous COVID‐19 vaccines have been proposed to prevent COVID‐19 disease, including the BNT162b2 (Pfizer–BioNTech), BBIBP‐CorV (Sinopharm), mRNA‐1273 (Moderna), ChAdOx1 nCoV‐19 (Oxford‐AstraZeneca), and Sputnik V vaccines.^3^ Some reports have pointed to the differences in immunogenicity levels and side effects of COVID‐19 vaccines in different populations.^3^, ^4^, ^5^, ^6^ Regarding that patients with immunodeficiency were excluded from the landmark Page 4/23 vaccines trials, the concern about adverse events of COVID‐19 vaccines in patients with AIIRD was proposed by the medical community. Today, several prospective controlled studies have provided stronger scientific evidence indicating SARS‐CoV‐2 vaccination was immunogenic in most patients with AIIRD, with an acceptable safety profile, although postvaccination level of anti‐SARS‐CoV‐2 immunoglobulin G (IgG) antibody was lower in patients with AIIRD than immunocompetent controls. In line with this notion, most studies pointing to the efficiency and side effects of COVID‐19 vaccination are the mRNA vaccines, such as BNT162b2, mRNA‐1273, and ChAdOx1 nCoV‐19 vaccines.^7^, ^8^, ^9^, ^10^ Previous studies have indicated that mRNA COVID‐19 vaccines had an acceptable safety and immunogenicity in the majority of patients with AIIRD.^10^, ^11^ However, some immunosuppressive drugs, such as glucocorticoids, rituximab, mycophenolate mofetil, and abatacept, can significantly reduce mRNA vaccine‐induced immunogenicity.^10^, ^12^ Furthermore, recent studies have revealed that different COVID‐19 vaccines resulted in no serious adverse events which required medical attention or hospitalization in patients with AIIRD.^13^ On the contrary, there are some evidence showing mRNA COVID‐19 vaccines (BNT162b2 and mRNA‐1273) failed to provide an effective response against SARS‐CoV‐2 infection in patients with AIIRD and the level of serum IgG‐neutralizing antibody was significantly lower in patients with AIIRD than healthy subjects.^14^

Despite numerous studies regarding short‐term immunogenicity and safety of Sinovac‐CoronaVac inactivated vaccine in patients with AIIRD,^11^, ^15^ we could not find any reports pointing to the efficiency and adverse events of other inactivated vaccines in these patients. In line with the immunogenicity of the inactivated vaccines, Sinovac‐CoronaVac vaccine provides a suitable immunogenicity in patients with AIIRD, although postvaccination level of anti‐SARS‐CoV‐2 IgG antibody is significantly lower than the mRNA COVID‐19 vaccines.^15^ Therefore, this study was focused on determining whether the BBIBP‐CorV, an inactivated SARS‐CoV‐2 vaccine, has acceptable efficiency and safety in patients suffering from autoimmunity, such as systemic lupus erythematosus (SLE), rheumatoid arthritis (RA), ankylosing spondylitis (AS), and systemic sclerosis (SSc).

## 2 MATERIALS AND METHODS

### 2.1 Study populations

This study included 100 AIIRD patients treated with immunosuppressive agents for several years before the vaccination and 100 healthy individuals without any history of health problems, malignancy, and autoimmunity, as a control group (Table 1). All AIIRD patients were recruited among those referred to the internal medicine clinic of Shahid Beheshti hospital, Kashan, Iran from June 2021 to April 2022. The diagnosis of AIIRD was approved by the internal medicine specialist according to American College of Rheumatology (ACR)‐European League Against Rheumatism (EULAR) criteria for RA,^16^ Systemic Lupus International Collaborating Clinics classification criteria for SLE,^17^ Spondyloarthritis International Society (ASAS) classification criteria for AS,^18^, ^19^ the 2013 ACR/EULAR classification criteria for SSc.^20^

**Table 1:** Demographic and clinical characteristics of participants vaccinated with the BBIBP‐CorV vaccine (*n* = 200).

	Healthy subjects (*n* = 100)	Patients with RA (*n* = 38)	Patients with SLE (*n* = 26)	Patients with SSc (*n* = 15	Patients with AS (*n* = 21)
Age, years, median (range)	39.47 ± 9 (19–63)	52.62 ± 11.98 (25–75)	49.93 ± 9.5 (35–65)	31.7 ± 6.7 (19–63)	42.15 ± 12.29 (21–65)
Gender, female, *n* (%)	56 (56%)	32 (84.2%)	24 (96%)	15 (100%)	4 (15.3%)
Smoking history	8 (8%)	4 (10.52%)	2 (8%)	0 (0.0%)	4 (15.3%)
Disease duration, years, median (range)	‐	8.2 ± 4.16 (3–18)	8.07 ± 3.8 (2–18)	6.5 ± 3.8 (4–14)	4.9 ± 2.3(3–10)
Background diseases	‐	Diabetes: 3 Hypertension: 6 Hyperlipidemia: 6 Other metabolic disorders: 1	Diabetes: 4 Hypertension:2 Hyperlipidemia: 2 Anemia: 1 Other metabolic disorders: 2	‐	Diabetes: 2 Other metabolic disorders: 1
Myalgia	14 (14%)	6 (15.7%)	7 (26.9%)	0 (0.0%)	3 (14.2%)
Fever	2 (0.0%)	2(5.23%)	2 (7.69%)	1 (6.6%)	3 (14.2%)
Headache	0 (0.0%)	4 (10.46%)	2 (7.69%)	0 (0.0%)	2 (13.3%)
Arthritis	0 (0.0%)	1 (2.61%)	0 (0.0%)	0 (0.0%)	0 (0.0%)
Skin lesions	0 (0.0%)	0 (0.0%)	1 (3.84%)	0 (0.0%)	0 (0.0%)
Concomitant immunosuppressive medications, *n* (%)					
Methotrexate	0 (0.0%)	30 (78.90%)	0 (0.0%)	12 (80%)	0 (0.0%)
Glucocorticoid	0 (0.0%)	38 (100%)	26 (100%)	14 (93.3%)	0 (0.0%)
Mycophenolate mofetil	0 (0.0%)	0 (0.0%)	23 (88.4%)	13 (86.6%)	0 (0.0%)
Hydroxyl chloroquine	0 (0.0%)	30 (78.90%)	24 (92.3%)	0 (0.0%)	0 (0.0%)
Sulfasalazine	0 (0.0%)	3 (7.8%)	4 (15.3%)	5 (33.3%)	18 (85.7%)
Anti‐tumor necrosis factor	0 (0.0%)	0 (0.0%)	3 (11.5%)	1 (6.6%)	20 (95.2%)

Abbreviations: AS, ankylosing spondylitis; RA, rheumatoid arthritis; SLE, systemic lupus erythematosus; SSc, systemic sclerosis.

All participants were negative for past vaccination allergy and pregnancy. None of the healthy subjects was on immunosuppressive treatments. This study was approved by the Ethics Committee of Kashan University of Medical Sciences (IR. KAUMS. MEDNT. REC.1400.148) and performed in accordance with the declaration of Helsinki. The informed consent was obtained from all participants before study initiation.

### 2.2 Vaccination procedure

All participants were received the two‐dose regimen of the BBIBP‐CorV vaccine based on national protocols. Some participants with a history of natural SARS‐CoV‐2 infection had an antibody level less than the threshold (2.5 µg/mL) for positive detection of SARS‐CoV‐2 neutralizing antibody at the time point before vaccination. Each 4‐μg dose of the BBIBP‐CorV vaccine was given as an intramuscular injection in the deltoid muscle, the second dose was given 4 weeks after the first dose.

### 2.3 The immunogenicity and safety of vaccination

To evaluate the immunogenicity of the BBIBP‐CorV vaccine, the serum samples (2 mL) of participants were collected before initial vaccination and 1–3 months after the second immunization. SARS‐CoV‐2 neutralizing antibodies were quantified using an enzyme‐linked immunosorbent assay kit (Ideal Tashkhis) according to the manufacturer's guidelines. Side effects were also assessed 7 days after the second dose. A questionnaire was used to the patients prospectively pertaining to side effects of vaccination.

### 2.4 Investigation of AIIRD activity

Disease activity assessment was carried out by a treating rheumatologist of the recruited patients and regained from the medical records within 12 weeks before immunization. Postvaccination AIIRD activity was studied 3–8 weeks after the second dose. Disease activity was assessed using Simplified Disease Activity Index, Clinical Disease Activity Index, and Disease Activity Score‐C‐reactive protein for RA,^21^ Systemic Lupus Disease Activity Index for SLE,^22^, ^23^ European Scleroderma Study Group to define disease activity criteria for SSc,^24^ and patient and physician global assessment for AS.^25^

### 2.5 Statistical analysis

Statistical analysis was carried out by GraphPad Prism 6 (GraphPad Software). The results are expressed as the mean ± standard deviation (SD) and mean ± standard error of the mean. The normal distribution of data was studied by Kolmogrov–Smirnov test. The groups with the normal distribution were compared by One‐way analysis of variance and unpaired *t*‐tests. Kruskal–Wallis and Mann–Whitney tests were employed to compare the groups with nonnormal distribution. The Pearson's and Spearman's tests were used to determine correlation coefficients of the data with normal and nonnormal distributions, respectively. The comparison of the ratios was studied using Chi‐square and Fisher's exact tests. *p* ≤ .05 were considered statistically significant.

## 3 RESULTS

### 3.1 Description of subjects

A total of 100 AIIRD subjects (mean age of 45 ± 14.15, mean ± SD, aged 18–74 years) participated in the study. Of the 100 AIIRD subjects, 38 had RA, 26 had SLE, 15 had SSc, and 21 had AS (Table 1). The most common adverse events of the BBIBP‐CorV vaccine among participants were fever, headache, and myalgia (Table 1). Table 1 depicts the clinical characteristics and other information of the AIIRD subjects.

### 3.2 The immunogenicity of BBIBP‐CorV vaccine

**Figure 1:**
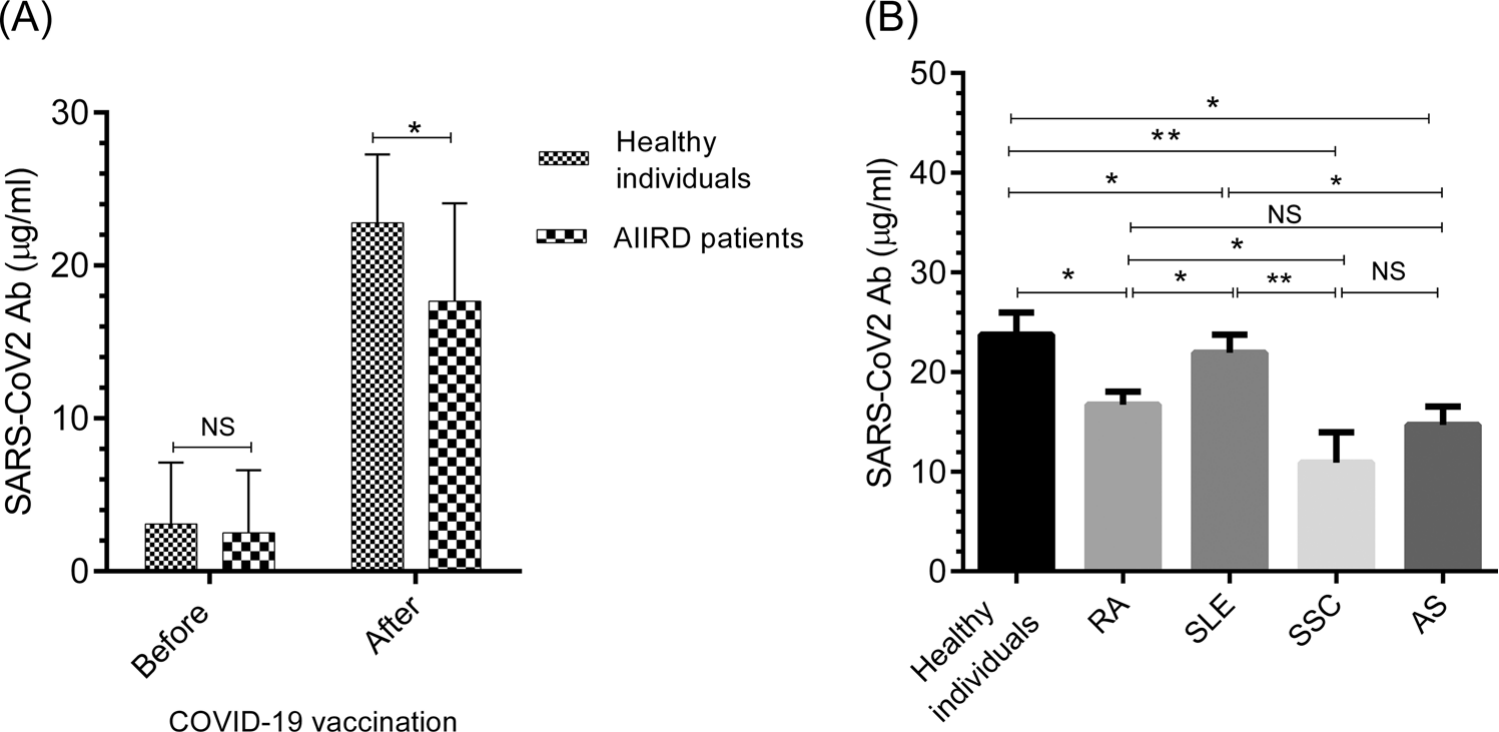
The immunogenicity of the BBIBP‐CorV vaccine in healthy and autoimmune inflammatory rheumatic diseases (AIIRD) subjects. SARS‐CoV‐2 neutralizing antibody titers in participants were studied by enzyme‐linked immunosorbent assay (ELIZA). (A) and (B) Each bar is representative of 100 independent experiments for patients with AIIRD (38 rheumatoid arthritis [RA] cases, 26 systemic lupus erythematosus [SLE] cases, 15 systemic sclerosis [SSc] cases, and 21 ankylosing spondylitis [AS] cases) and 100 independent experiments for healthy subjects. All data show mean ± standard deviation (SD). NS (non significant) indicates that the difference in the expression levels is not statistically significant. Kruskal–Wallis and Mann–Whitney tests were used to compare the groups with nonnormal distributions. *p < .05, **p < .01.

The values of SARS‐CoV‐2 neutralizing antibodies were assessed to determine the efficiency of the BBIBP‐CorV vaccine. Vaccination‐induced positive immunogenic response rates and serum SARS‐CoV‐2 neutralizing antibody titers were significantly lower in the AIIRD patients than healthy subjects, 60.5% versus 72.8% and 17.58 ± 20.2 µg/mL versus 22.87 ± 20.9 µg/mL, respectively (*p* < .05, Figure 1A). Other results indicated that there are significant differences in the values of neutralizing antibodies among patients suffering from RA, SLE, SSc, and AS (*p* < .01–.05, Figure 1B). The lowest neutralizing antibody titers were respectively detected in patients with AS and SSc, 10.88 ± 8.16 and 14.69 ± 7.11, whereas the highest values were observed in patients with SLE and RA, 21.91 ± 9.87 and 16.71 ± 8.29, respectively (Figure 1B). The rates of seropositive vaccine responses were similarly distributed across all diseases, which were 68.3%, 73.4%, 71.5%, and 73.4% for RA, SLE, SSc, and AS, respectively (Table 2).

**Table 2:** Adverse events as documented 7 days after secondary immunization in healthy and patient groups.

	Healthy subjects (*n* = 100)	Patients with RA (*n* = 38)	Patients with SLE (*n* = 26)	Patients with SSc (*n* = 15)	Patients with AS (*n* = 21)
Fever ≥38.0°C, *n* (%)	12 (12%)	1 (2.63%)	2 (7.5%)	3 (20%)	4 (23.8%)
Myalgia, *n* (%)	8 (8%)	7 (19.7%)	5 (20.9%)	0 (0.0%)	4 (23.8%)
Headache, *n* (%)	14 (14%)	6 (17.78%)	2 (7.5%)	0 (0.0%)	2 (13.3%)
Joint swelling, *n* (%)	0 (0.0%)	1 (2.63%)	0 (0.0%)	0 (0.0%)	0 (0.0%)
Shortness of breath, *n* (%)	0 (0.0%)	1 (2.63%)	0 (0.0%)	0 (0.0%)	0 (0.0%)
Hypoglycemia, *n* (%)	0 (0.0%)	1 (2.63%)	0 (0.0%)	0 (0.0%)	0 (0.0%)
Skin lesions, *n* (%)	0 (0.0%)	0 (0.0%)	1 (3.84%)	0 (0.0%)	0 (0.0%)
Gastrointestinal disorders, *n* (%)	1 (1%)	0 (0.0%)	0 (0.0%)	0 (0.0%)	0 (0.0%)
Lethargy, *n* (%)	1 (1%)	0 (0.0%)	0 (0.0%)	0 (0.0%)	0 (0.0%)

Abbreviations: AS, ankylosing spondylitis; RA, rheumatoid arthritis; SLE, systemic lupus erythematosus; SSc, systemic sclerosis.

### 3.3 The BBIBP‐CorV vaccine safety in healthy and AIIRD individuals

Healthy and AIIRD individuals had a similar profile in side effects and their rates were 30% versus 34.3%, in healthy and AIIRD subjects, respectively. Other results revealed that there is no significant difference in SARS‐CoV‐2 antibody values between subjects who suffered from vaccine‐related adverse events and those who did not have. Table 2 indicates vaccine‐related adverse events in healthy and AIIRD subjects.

### 3.4 Vaccine immunogenicity and reactogenicity in SARS‐CoV‐2 seropositive and seronegative individuals

**Figure 2:**
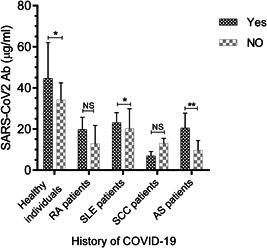
Vaccine immunogenicity in SARS‐CoV‐2 seropositive and seronegative individuals. SARS‐CoV‐2 neutralizing antibody levels in seropositive and seronegative persons were studied by enzyme‐linked immunosorbent assay (ELIZA). The depicted results are representative of 57 independent experiments for seropositive subjects and 49 independent experiments for seronegative subjects. All data show mean ± standard deviation (SD). NS indicates that the difference in the expression levels is not statistically significant. Mann–Whitney and unpaired *t*‐tests were used to compare two groups with nonnormal and normal distributions, respectively. *p < .05, **p < .01.

The efficiency of the BBIBP‐CorV vaccine was studied in SARS‐CoV‐2‐noninfected and‐infected persons. The rates of seropositive vaccine responses in healthy and AIIRD subjects previously infected with SARS‐CoV‐19 were 87.5% versus 76%, respectively. While, these rates in healthy and AIIRD subjects who did not have a history of natural SARS‐CoV‐2 infection were 85% versus 63.8%, respectively. The serum levels of SARS‐CoV‐2 neutralizing antibodies were significantly higher in healthy and AIIRD patients with a history of SARS‐CoV‐2 infection than seronegative subjects, with the exception of patients with SSc (Figure 2, *p* < .01–.05).

In the next step, the reactogenicity of vaccination was studied in seropositive and seronegative subjects. We observed that the percentages of vaccine‐related adverse events were significantly higher in seropositive individuals than those without a history of COVID‐19 (*p* < .05, 95% confidence interval: 0.2490–0.9543, odds ratio: 0.3660).

### 3.5 BBIBP‐CorV vaccine effects on disease activity in the AIIRD patients

Disease activity indices were overall stable in all patients, although there was a negative impact on disease activity in patients with RA and SLE.

## 4 DISCUSSION

The immunogenicity and side effects of COVID‐19 vaccines in the AIIRD patients remains debatable. Herein, the main aim of the current study was to assess whether patients with AIIRD requiring immunomodulatory treatments can mount a positive serologic response to the BBIBP‐CorV vaccine against SARS CoV‐2. We studied the immunogenicity and adverse events of vaccination in the AIIRD patients vaccinated with the two‐dose BBIBP‐CorV vaccine regimen. Postvaccination AIIRD activity was also evaluated.

In our knowledge, there is no data demonstrating the capability of the BBIBP‐CorV vaccine in induction of an immunogenic serologic response in patients with AIIRD. Until now, several studies have pointed to the immunogenicity and side effects of mRNA vaccines against SARS‐CoV‐2 in the AIIRD patients.^2^, ^26^ Our results revealed that the AIIRD patients had an acceptable titer of neutralizing antibody against SARS‐CoV‐2 infection. However, the rate of seropositive vaccine response and SARS‐CoV‐2 neutralizing antibody level were significantly lower in the AIIRD patients than healthy subjects, 60.5% versus 72.8%. Other results indicated that the rates of seropositive vaccine responses were similarly distributed across all diseases, despite the observed differences in the values of neutralizing antibodies among patients suffering from RA, SLE, SSc, and AS. Moreover, the frequencies of adverse events were similar in healthy and AIIRD subjects. No significant difference was observed in the values of SARS‐CoV‐2 antibodies between subjects suffering from vaccine‐related adverse events and those who did not have. Although some reports have pointed that mRNA SARS‐CoV‐2 vaccines failed to induce an acceptable immune responses in patients with rheumatic diseases under immunotherapies,^27^ our findings are consistent with previous studies showing inactivated CoronaVac and SARS‐CoV‐2 mRNA vaccines are immunogenic in the majority of patients with AIIRD.^10^ In this regard, there is a decreased antibody response in the AIIRD patients compared to the healthy control group.^2^, ^10^, ^11^ Previous studies have shown that CoronaVac vaccine, with an acceptable safety profile, can lead to the development of antibodies in patients with chronic inflammatory conditions, although the seroconversion rate was slightly lower in the patients received inactivated vaccine than those vaccinated with the BNT162b2 mRNA vaccine.^11^ Several lines of evidence indicate that the mRNA COVID‐19 vaccines had significant immunogenicity in healthy and AIIRD subjects without considerable side effects or induction of disease flares.^10^ Furthermore, it is reported that mRNA vaccines have the immunogenicity in the majority of patients with AIIRD, which is lower than healthy individuals.^10^, ^26^ Our data aligns with other studies conducted on the AIIRD patients vaccinated with the BNT162b2 mRNA vaccine suggesting that the reduced levels of SARS‐CoV‐2 neutralizing antibodies may relate to the negative impacts of immunosuppressive agents on vaccine‐induced immunogenicity.^2^, ^7^, ^10^

In the next step, vaccine immunogenicity was studied in seropositive and seronegative persons. Our data indicated that the rates of seropositive vaccine responses and serum levels of SARS‐CoV‐2 neutralizing antibodies were significantly higher in healthy and AIIRD patients with prior history of natural SARS‐CoV‐2 infection than seronegative subjects, with the exception of patients with SSc. In addition, seropositive people experienced a significant increase in vaccine‐associated side effects. In agreement with these observations, some reports have shown that a single dose of mRNA vaccine elicited rapid immune responses in seropositive subjects, which were similar to or exceeded titers observed in participants without pre‐existing immunity who received two‐dose of the mRNA vaccines.^28^ In line with this notion, others have indicated that the BNT162b2 SARS‐CoV‐2 vaccine was able to improve IgG level in both individuals with and without pre‐existing immunity; however, only seropositive individuals had increased IgA or IgM antibody titers after a single dose.^29^ These findings propose that exceeded antibody titers in subjects with a history of COVID‐19 may correlate to the acquired immune system and immune memory persistence, developed after the viral exposure. Therefore, antibody assessment before vaccination is required to provide an acceptable immunogenic response against SARS‐CoV‐2 infection.

In an effort to determine the safety of the BBIBP‐CorV vaccine in seronegative and seropositive persons, our data revealed that vaccine recipients with pre‐existing immunity had a significant increase in vaccine‐related side effects compared to individuals without pre‐existing immunity. In agreement with this observation, others have demonstrated that seropositive subjects had systemic side effects at higher frequencies than those without a history of COVID‐19.^28^ In line with adverse events of COVID‐19 vaccines, a study conducted by Efrati et al. it was showed that the BNT162b2 mRNA vaccine could result in short‐term severe symptoms that required medical attention in subjects previously infected with SARS‐CoV‐19, while none were found in seronegative individuals.^30^ The higher frequencies of side effects of vaccination in postinfected patients may correlate to over activation of the immune system or allergic reactions, which sometimes need to medical supervision and care.

Regarding that another aim of the current study was the investigation of impact(s) of the BBIBP‐CorV vaccine on AIIRD activity, postvaccination disease activity indices were assessed. As mentioned above, vaccination did not have a significant effect on disease flares in patients with AIIRD. We observed that the rheumatic disease remained stable in the majority of patients, which is consistent with the observed outcome of SARS‐CoV‐2 mRNA vaccination on patients with AIIRD.^2^, ^7^, ^10^, ^31^ It is reported that the BNT162b2 mRNA vaccine did not have an apparent impact on AIIRD activity.^7^ Further evidence to support this finding is a study on Greek patients with systemic autoimmune and autoinflammatory rheumatic diseases showing mRNA SARS‐CoV‐2 vaccines are effective and safe in the induction of anti‐SARS‐CoV‐2 antibody responses without affecting disease flares.^32^

Taken together, vaccination with the BBIBP‐CorV vaccine results in an acceptable immunogenic response, which is safe, in terms of adverse events and disease flares, in the majority of patients with AIIRD. Our results can provide reassurance to the AIIRD patients treated with immunomodulatory drugs and their physicians, regarding the short‐term immunogenicity and safety of the BBIBP‐CorV vaccine. However, the present study has several limitations. The relatively small sample size of patients with RA, SLE, SSc, and AS which may not represent the overall population. In addition, reactogenicity could be investigated after the first dose. Healthy and patient groups were not similar with regard to age, gender, and concomitant immunosuppressive medications use. This study investigated COVID‐19 postinfected subjects who had relatively low pre‐vaccination antibody levels. Finally, cell‐mediated immunity responses were not studied, which can provide further evidence regarding immune responses, especially in seronegative individuals with a history of natural SARS‐CoV‐2 infection. Therefore, it should be noted that these limitations would be considered to confirm our findings in future studies.

## AUTHOR CONTRIBUTIONS

**Ahmad Piroozmand**: Investigation; methodology. **Mahsa Dehghani**: Methodology; visualization. **Maryam Arfaatabar**: Formal analysis; investigation.

## CONFLICT OF INTEREST STATEMENT

The authors declare no conflict of interest.

## ETHICS STATEMENT

The study was approved by the ethics committee of Kashan University of Medical Sciences (IR. KAUMS. MEDNT. REC.1400.148). Informed consent was taken before taking part in the study. All authors agree to publish the article.

## Data Availability

All data generated or analyzed during this study are included in this manuscript.
